# Creativity in high-fidelity simulation-based nursing education: associations with learning outcomes and stress

**DOI:** 10.1186/s12912-026-04751-4

**Published:** 2026-05-15

**Authors:** Agata Wojcieszek, Anna Kurowska, Aldona Wróbel, Iwona Bodys-Cupak, Alicja Kamińska, Anna Majda

**Affiliations:** Department of Theory and Fundamentals of Nursing, Institute of Nursing and Midwifery, Faculty of Health Sciences, Jagiellonian University Medical College, Krakow, Poland

**Keywords:** Nursing education, Creativity, High-fidelity simulation, Nursing students, Stress, Learning outcomes

## Abstract

**Introduction:**

Contemporary nursing education increasingly relies on evidence-informed teaching strategies that improve learning outcomes while supporting students’ psychological well-being. High-fidelity simulation (HFS) is a well-established educational method; however, less is known about the role of individual learner characteristics, such as creativity, in shaping its effectiveness.

**Aim of the study:**

This study aimed to examine creativity in the cognitive and characterological domains and to analyze its relationship with satisfaction, self-confidence, perceived learning outcomes, and stress experienced during high-fidelity simulation classes among nursing students.

**Materials and methods:**

A cross-sectional study was conducted among 286 first-year undergraduate nursing students participating in HFS classes during the 2022/2023 and 2023/2024 academic years. Data were collected using an author-designed questionnaire, the Student Satisfaction and Self-Confidence in Learning Scale (SSCL), and the Creative Behavior Questionnaire (KAHN III). Statistical analysis included Spearman’s rank correlation coefficient and the Mann–Whitney U test, with the level of significance set at *p* < 0.05.

**Results:**

Higher levels of creativity in both the cognitive and characterological domains were positively associated with students’ satisfaction, self-confidence, and selected perceived learning outcomes. Characterological creativity was positively associated with perceived development of knowledge, practical skills, and social competencies, whereas cognitive creativity was associated with perceived development of knowledge and social competencies. Higher creativity was also associated with lower levels of stress related to independent task performance, patient communication, and procedural execution during simulation scenarios.

**Conclusions:**

The findings suggest that creativity may be an important learner-related factor associated with more favorable educational experiences in high-fidelity simulation. Higher creative dispositions were linked to greater satisfaction and self-confidence, as well as lower stress during simulation activities. These results support the inclusion of creativity-supportive elements in the design of nursing simulation curricula.

**Trial registration:**

Not applicable.

**Supplementary Information:**

The online version contains supplementary material available at 10.1186/s12912-026-04751-4.

## Introduction

Creativity should be considered a valuable, though often overlooked, attribute in the education of healthcare professionals. Although clinical skills training has traditionally relied on standardized patterns and algorithms, contemporary health professions education increasingly emphasizes active, experience-based approaches that foster problem-solving, reflection, and learner engagement. Within this context, simulation-based education, particularly high-fidelity simulation (HFS), offers an opportunity not only to practice technical procedures in a safe environment, but also to develop communication, teamwork, decision-making, and adaptive responses to complex clinical situations. In simulated settings, creativity may support the generation of context-appropriate solutions to complex clinical problems [1–4].

In addition to generating new and useful ideas, creativity also has an adaptive dimension. Previous reports have suggested that higher creativity may be associated with more positive emotions, lower perceived stress, and better coping in adverse circumstances [5]. Creativity may therefore be understood not only as a dispositional characteristic, but also as a psychological resource that facilitates adaptation to changing external conditions. Moreover, creativity should not be understood exclusively as a fixed, innate trait; although it includes relatively stable dispositional components, its expression may be fostered by educational environments and targeted learning experiences [6–8]. Accordingly, theoretical models suggest that creativity depends not only on individual characteristics, but also on contextual factors, motivation, and opportunities for active engagement [9].

Creativity, understood as a multidimensional human capacity to create, requires the activation of cognitive resources, motivational-emotional capacities, and personality-related conditions. The interplay of these factors may contribute to goal attainment and to the development of an individual’s potential. An important conceptualization of creativity was proposed by Popek, who defined it as a continuous readiness to explore and transform both reality and one’s own personality. According to this concept, such readiness is expressed through two interrelated spheres: the cognitive sphere and the characterological sphere. The cognitive sphere encompasses intellectual dispositions that form algorithmic or heuristic behaviors, whereas the characterological sphere includes personality traits expressed along the conformity-nonconformity continuum. In Popek’s view, a creative attitude results from the configuration of nonconformity and heuristic behaviors, while a reproductive attitude reflects conformity and algorithmic behaviors [10].

The relevance of creativity in simulation-based learning may also be understood through broader theoretical perspectives. From a cognitive perspective, creativity involves flexible thinking, problem-solving, and the ability to generate and adapt responses to novel situations. These capacities appear particularly relevant in simulation-based learning, where students must interpret clinical cues, integrate prior knowledge, and respond to evolving scenarios [11]. From a socio-constructivist perspective, both learning and creativity are shaped through interaction, dialogue, and engagement in meaningful tasks. This assumption is especially relevant in simulation-based education, where students learn not only through individual action, but also through teamwork, observation, discussion, and reflective debriefing [12]. Experiential learning theory also offers a useful lens for understanding the role of creativity in HFS, as simulation provides concrete experience followed by reflection, conceptualization, and active experimentation, which may promote adaptive thinking and the development of more flexible responses in future clinical situations [13]. This interpretation is also consistent with simulation-based learning frameworks such as the NLN Jeffries Simulation Theory, which emphasizes that simulation outcomes are shaped by the interaction between design characteristics, facilitation, educational practices, and learner-related factors. Within this framework, creativity may be viewed as one of the learner characteristics influencing how students experience and benefit from simulation [14]. Taken together, these perspectives suggest that creativity may be understood not only as an individual disposition, but also as a learner-related resource expressed and developed in interaction with educational design, clinical complexity, and reflective practice.

The simulated environment in HFS is often complex and requires participants to generate responses that go beyond routine patterns. The 2018 report by the Organisation for Economic Co-operation and Development (OECD), *Learning Framework 2030*, identified creativity as an essential competence for learners and recommended it as an important area of education [15]. In preliminary research conducted by Jackson et al., it was suggested that medical students need to be creative in order to make clinical decisions, solve problems, apply technical skills, and communicate with patients in a thoughtful and empathetic manner [16]. Another study found that nurses who think critically and creatively may be better prepared to cope with the diversity and complexity of the nursing environment, enhance patient safety, and improve treatment outcomes [17]. Moreover, nurses frequently encounter unpredictable situations for which they were not previously trained and must therefore demonstrate not only competence and professional attitude, but also the ability to respond in a creative and innovative way [18]. In nursing practice, creativity should not be understood merely as originality, but as a resource that may support safe and adaptive functioning in complex clinical situations. Nurses are required to make decisions under conditions of uncertainty, respond to unexpected changes in patients’ conditions, communicate effectively with patients and team members, and adjust their actions to dynamic care environments. In this sense, creativity does not stand in opposition to protocol-based care, but may complement it by supporting flexible, context-appropriate, and safe responses when clinical situations do not fully match routine patterns.

Although creativity has been widely discussed in educational research, direct assessment of creativity in adult learners remains relatively uncommon [19]. The present study is part of a broader research project exploring educational and psychological factors associated with high-fidelity simulation in undergraduate nursing education [20]. Although HFS has been widely examined in relation to outcomes such as stress, satisfaction, self-confidence, and perceived learning effectiveness [21, 22], less is known about the role of creativity as a learner-related resource in this context. Given that the educational effects of simulation may depend not only on scenario design and facilitation, but also on individual learner characteristics [23], clarifying whether creativity is associated with students’ experiences in HFS may be relevant not only for theory, but also for the pedagogical design of simulation-based nursing education, particularly if creativity can be supported through simulation design and facilitation. Therefore, the present cross-sectional study was designed to assess the level of creativity among first-year undergraduate nursing students and to examine the relationships between its cognitive and characterological dimensions and students’ opinions on simulation classes, satisfaction, self-confidence, and stress experienced during simulation scenarios. We assumed that examining creativity in the context of HFS would help explain individual differences in students’ experiences during simulation. The literature suggests that creativity is a trait distributed across the population and naturally varies from low to high levels [24]. However, in average individuals, its development may depend on environmental and social factors [25]. Based on this, it was assumed that the surveyed students would predominantly exhibit a conformist attitude with a tendency toward algorithmic behaviors. In addition, positive personality activation, understood as a specific combination of nonconformist traits and heuristic tendencies that stimulate creative potential in the cognitive and behavioral spheres, was expected to be associated with greater satisfaction, higher self-confidence in learning, and more favorable evaluations of knowledge, practical skills, and social competencies. Furthermore, it was assumed that a higher level of creativity could function as a protective factor in the stressful context of simulation.

## Materials and methods

### Research design and data collection

An anonymous cross-sectional study was conducted among first-year full-time undergraduate nursing students between May 2023 and June 2024. The study included students enrolled in the 2022/2023 and 2023/2024 academic years. The inclusion criteria were enrollment in a bachelor’s nursing program and active participation in high-fidelity simulation (HFS) classes as part of the “Basics of Nursing” course. A convenience sample of eligible first-year undergraduate nursing students was recruited from two consecutive academic cohorts. No a priori sample size calculation was performed. The sample size was determined by the number of eligible students available during the study period who met the inclusion criteria and participated in mandatory HFS classes. A total of 299 students were invited to participate, 289 agreed, and data from 286 participants were included in the final analysis due to incomplete responses in three cases. Thus, the final sample reflected the maximum accessible population within the defined time frame and setting.

Students participated in HFS sessions in 6-person groups, with each scenario performed by a 2-person team. Thus, during any given scenario, four students functioned as observers. Their role was to follow predefined learning objectives, observe the performance of their peers, and participate in the structured debriefing. Because all students rotated through the three scenarios within their group, each participant had the opportunity to actively perform in one scenario and to observe in the remaining scenarios. The questionnaires were completed after the completion of all simulation sessions; therefore, all participants responded on the basis of both active and observational engagement in HFS, and the data were analyzed jointly for the whole study group.

For the purposes of the study, urinary catheterization simulation scenarios were developed by the teaching team. The final three scenarios were selected to represent a comparable level of difficulty and different clinical indications for the procedure, including postoperative urinary retention, catheterization for diagnostic purposes, and fluid balance monitoring in a patient with circulatory failure. The scenarios were aligned with first-year nursing learning outcomes in the domains of knowledge, practical skills, and social competencies. An auxiliary checklist was also prepared to support instructors during scenario facilitation and debriefing. During the scenarios, students were required not only to perform the procedure itself, but also to demonstrate knowledge of the indications and contraindications for catheterization, therapeutic communication with the patient or family members, and effective communication within the therapeutic team.

All scenarios were standardized in terms of learning objectives, overall structure, instructor prompts, and expected student actions, ensuring comparable educational exposure across sessions. The sessions were conducted using the SimMan 3G high-fidelity patient simulator, which allowed the recreation of a realistic clinical environment and supported scenario-based learning in a controlled simulation setting. Each HFS class lasted 180 min in total (4 academic hours of 45 min each). Within this time, students completed three scenarios, preceded by pre-debriefing and followed by structured debriefing. The instructors conducting the classes had undergone prior training in the facilitation of HFS-based sessions, which supported the uniform delivery of the exercises according to the established plan.

After completion of the sessions at the multidisciplinary Center for Innovative Medical Education (CIEM), members of the research team who were not involved in conducting the classes provided students with detailed information about the study. Participants were informed about the aim and procedure of the study, the anonymous and voluntary nature of participation, and the possibility of withdrawing at any stage without giving a reason and without any consequences for their studies or evaluation. Data collection was conducted after completion of all simulation sessions and was carried out by research team members not involved in teaching, thereby minimizing potential instructional bias. Students who agreed to participate received printed research tools together with a Participant Information Sheet. After completing the forms, they placed them in a sealed box located in the simulation center. No financial or material compensation was provided. Potentially identifiable data were treated as confidential in accordance with applicable legal regulations. The collected data were stored in a secure location on a password-protected computer with an encrypted hard drive, and access was restricted to the project manager only. The data will be retained for no longer than 5 years after completion of the study and data analysis.

The study was designed, conducted, and reported in accordance with the principles of Good Scientific Practice and the Helsinki Declaration. Approval was obtained from the Research Ethics Committee of the Jagiellonian University Medical College (decision no. 118.6120.28.2022). The study was funded under the statutory project no. N43/DBS/000253.

### Instruments

Data were collected using an author-designed questionnaire and two standardized instruments: the Student Satisfaction and Self-Confidence in Learning Scale (SSCL) and the Creative Behavior Questionnaire (KAHN III).

- **Author-Designed Questionnaire**: The author-designed questionnaire was developed specifically for the purposes of the present study to assess students’ opinions on the simulation sessions, including perceived competence development, attractiveness of the classes, stress experienced during the simulation, and selected elements of debriefing. It consisted of 15 single-choice questions rated on a 5-point Likert scale (strongly disagree = 1, disagree = 2, neither agree nor disagree = 3, agree = 4, strongly agree = 5). The questionnaire also included demographic questions regarding sex, age, and academic year. As the instrument was developed for this study, it did not undergo full psychometric validation, which is acknowledged as a limitation [see Additional file 1].
- **Satisfaction and Self-Confidence of Students in the Learning Process (SSCL)**: The scale was developed by Pamela R. Jeffries and Mary Anne Rizzolo. The tool consists of two subscales designed to assess personal feelings and satisfaction with the simulation. The subscales measure students’ satisfaction with the learning process (five statements) and their self-confidence in the learning process (eight statements). In total, the scale consists of thirteen statements. Each statement could be rated on a five-point scale. The reliability calculated using Cronbach’s Alpha coefficient was 0.87 for satisfaction and 0.84 for self-confidence in the Polish version of the tool, which was developed and validated by Katarzyna Studnicka, Danuta Zarzycka, and Jakub Zalewski [26, 27].
- **Creative Behavior Questionnaire (KAHN III)** – The theoretical foundations of this tool are based on the works of S. Popek. KAHN III consists of two scales: the Conformity-Nonconformity (C-N) scale, which belongs to the personality domain (statements: 2, 4, 5, 7, 9, 12, 16, 18, 20, 22, 24, 25, 26), and the Algorithmic-Heuristic Behavior (A-H) scale (statements: 1, 3, 6, 8, 10, 11, 13, 14, 15, 17, 19, 21, 23), which falls under the cognitive domain. Each scale assesses 13 traits arranged along a continuum. The questionnaire consists of 26 statements in total. Responses are scored according to the following key: “yes” – 4 points; “rather yes” – 3 points; “no opinion” – 2 points; “rather no” – 1 point; “no” – 0 points. Exceptions are questions 15 and 24, for which the scoring should be reversed for the answers (except for “C” – 2 points, which remains unchanged). The reliability of the tool, measured by the test-retest method, is 0.95. The reliability calculated by the scale’s authors (*N* = 4271) using Cronbach’s alpha for the C-N scale was 0.69, and for the A-H scale, it was 0.69 [7, 28]. In the present study, the internal consistency of the KAHN III subscales, assessed with Cronbach’s alpha, was 0.687 for the C-N scale and 0.729 for the A-H scale.

### Data analysis

The analysis of quantitative variables (i.e., numerical data) was performed by calculating descriptive statistics such as mean, standard deviation, median, quartiles, minimum, and maximum values. Correlations between quantitative variables were analyzed using Spearman’s correlation coefficient. The comparison of quantitative variables between two groups was conducted using the Mann-Whitney U test. A significance level of 0.05 was adopted for the analysis. The analysis was performed using R software, version 4.4.1 [29].

## Results

Data from 286 students were analyzed. There were significantly more women (*N* = 264; 92.30%) than men (*N* = 22; 7.70%). The students ranged in age from 20 to 22 years.

The average score on the C-N scale was 31.64 points (SD = 6.24), and for the A-H scale, it was 32.86 points (SD = 6.46). No differences in the level of creativity between the academic years were observed (*p* > 0.05) (Table 1).

**Table 1:** Distribution of participants’ responses on the C-N and A-H scales, including differences between academic years

KAHN III	Academic year	*N*	M	SD	Me	Min	Max	Q1	Q3	*p*
C-N scale	2022/2023	153	31.32	6.2	32	17	48	27	35	*p* = 0.508
	2023/2024	133	32	6.28	32	18	47	27	37	
A-H scale	2022/2023	153	32.15	6.25	32	13	46	28	36	*p* = 0.093
	2023/2024	133	33.67	6.62	34	16	48	29	39	

**p** - Mann-Whitney U test, **N** - number of observations, **M** - mean, **SD** - standard deviation, **Me** - median, **Min** - minimum, **Max** - maximum, **Q1** - first quartile, **Q3** - third quartile, **p** - statistical value, **C-N** - conformity-nonconformity scale, **A-H** - algorithmic-heuristic behaviors scale, Source: authors’ analysis

**Fig. 1:**
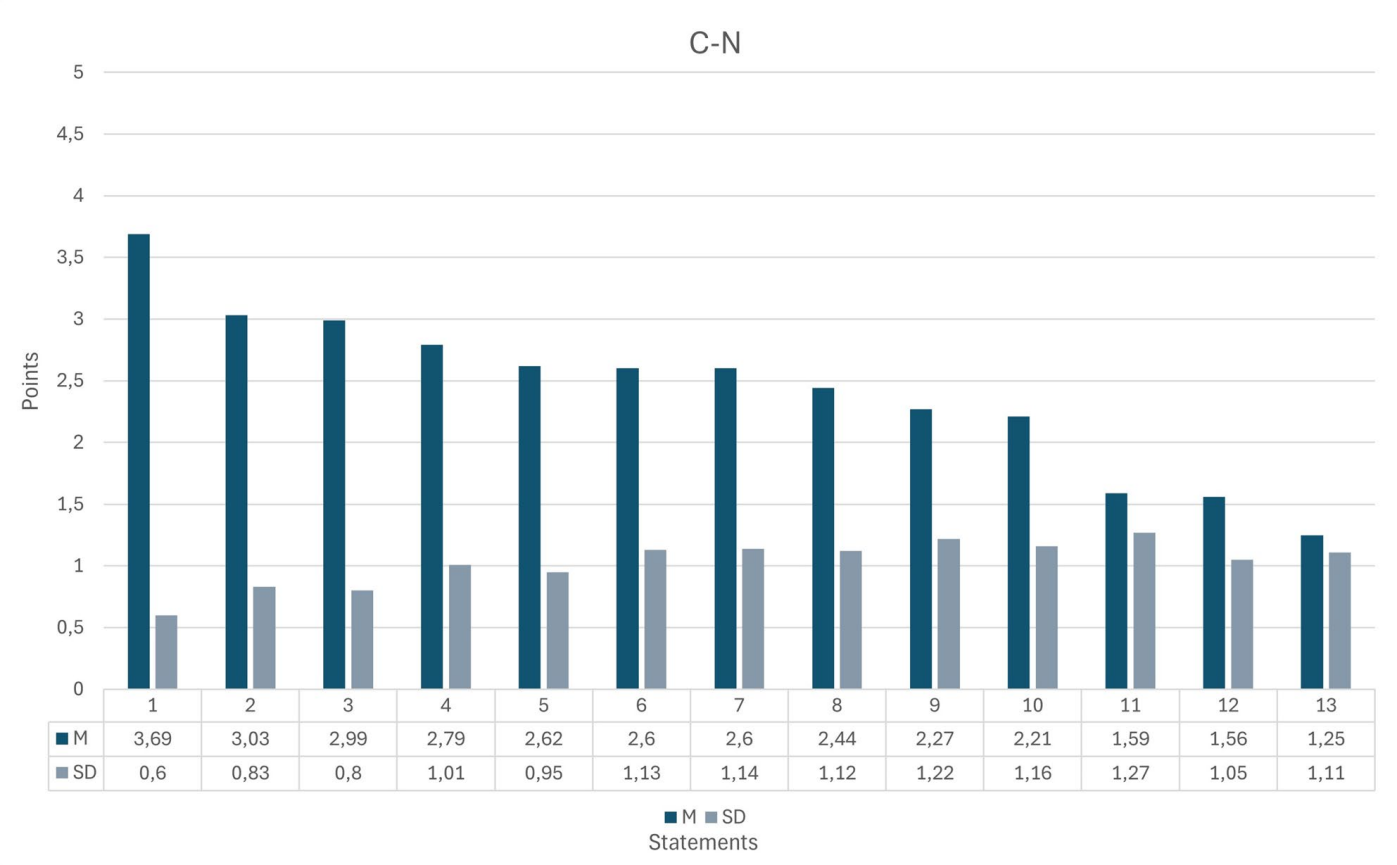
Conformity–non-conformity (C-N) dimensions of creative attitude. Mean (M) scores and standard deviations (SD) for 13 bipolar personality dimensions describing conformity–non-conformity orientations. The dimensions range from conformity and a reproductive attitude (C) to non-conformity and a creative attitude (N). The dimensions include: (1) intolerance (C) vs. tolerance (N); (2) lack of criticism (C) vs. self-criticism (N); (3) submissiveness (C) vs. consistency (N); (4) defensiveness (C) vs. openness (N); (5) low resilience and perseverance (C) vs. resilience and perseverance (N); (6) intellectual stiffness (C) vs. adaptational flexibility (N); (7) passiveness (C) vs. activeness (N); (8) dependence (C) vs. independence (N); (9) subordination (C) vs. dominance (N); (10) reliance (C) vs. self-organisation (N); (11) timidity (C) vs. courage (N); (12) stereotypicality (C) vs. originality (N); and (13) low self-esteem (C) vs. high self-esteem (N). Higher scores indicate a more creative (non-conformist) orientation

The highest intensity of creative attitudes was observed in statements related to tolerance (M = 3.69; SD = 0.60), self-criticism (M = 3.03; SD = 0.83), and consistency (M = 2.99; SD = 0.80). Conversely, the highest intensity of reproductive attitudes was noted in the areas of fearfulness (M = 1.59, SD = 1.27), stereotypicality (M = 1.56, SD = 1.05), and low self-esteem (M = 1.25, SD = 1.11). Similarly, the greatest intensity of heuristic behaviors was evident in learning through understanding (M = 3.35, SD = 0.66), reflectiveness (M = 3.14, SD = 0.81), and divergent thinking (M = 3.10, SD = 0.72) (Fig. 1).

**Fig. 2:**
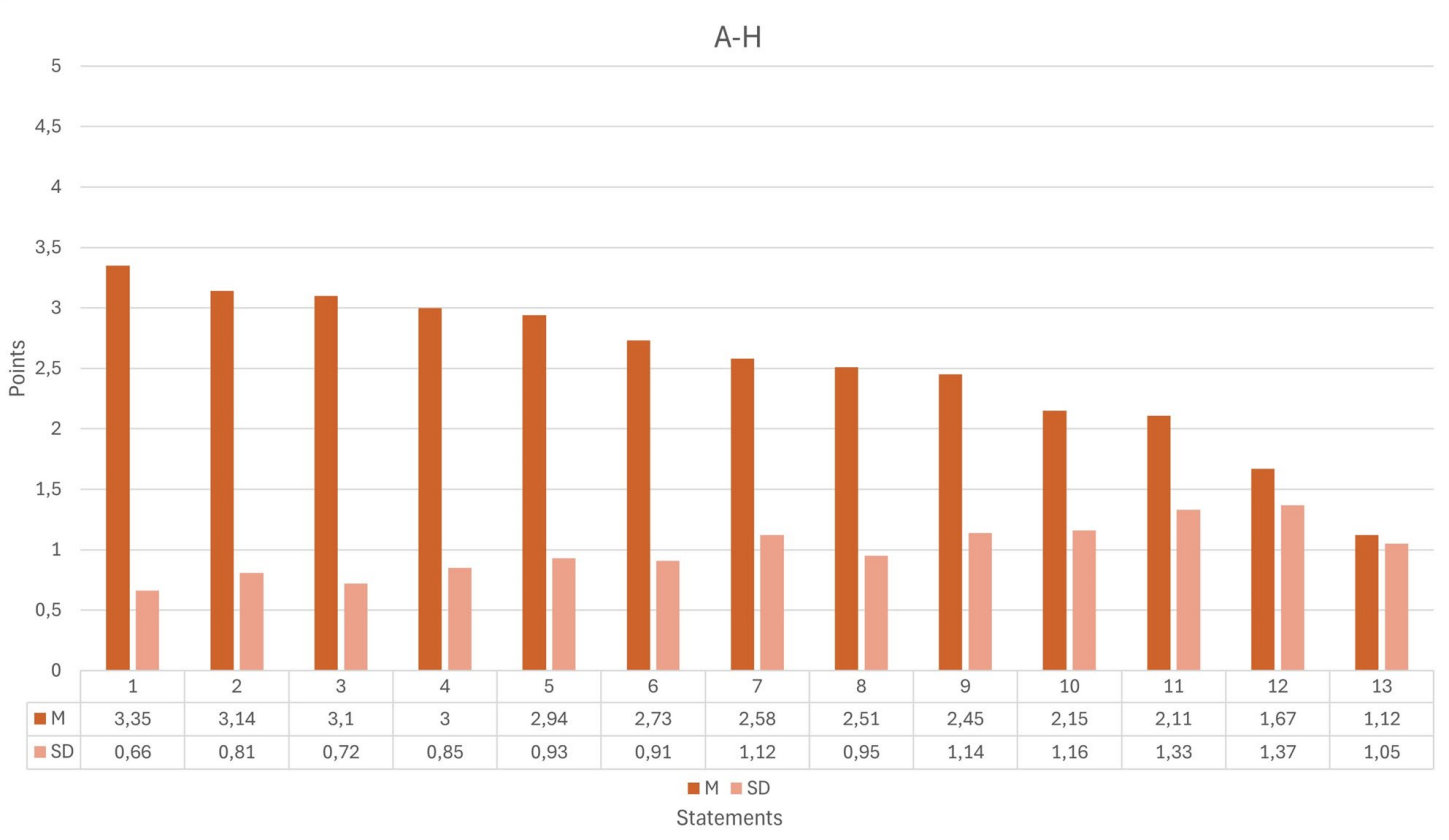
Creative–reproductive learning preferences across algorithmic–heuristic (A–H) dimensions. Mean (M) scores and standard deviations (SD) for 13 bipolar dimensions describing learning preferences, ranging from algorithmic/reproductive (A) to heuristic/creative (H) behaviour. The dimensions include: (1) learning through reasoning (A) vs. learning through understanding (H); (2) low reflectivity (A) vs. high reflectivity (H); (3) convergent thinking (A) vs. divergent thinking (H); (4) guided perceptiveness (A) vs. independence of observation (H); (5) cognitive passiveness (A) vs. cognitive activeness (H); (6) intellectual stiffness (A) vs. intellectual flexibility (H); (7) imitative imagination (A) vs. creative imagination (H); (8) copying (A) vs. intellectual self-reliance (H); (9) lack of technical ingenuity (A) vs. technical ingenuity (H); (10) low constructional skill and aptitude (A) vs. constructional skill and aptitude (H); (11) lack of artistic aptitude (A) vs. artistic aptitude (H); (12) verbal imitativeness (A) vs. verbal creativity (H); and (13) guided learning (A) vs. independent learning (H). Higher scores indicate a more creative (heuristic) learning orientation

In contrast, the highest intensity of algorithmic behaviors was expressed through a lack of artistic skills (M = 2.11; SD = 1.33), verbal reproduction (M = 1.61, SD = 1.37), and directed learning (M = 1.12, SD = 1.05) (Fig. 2).

The score on the C-N scale correlated significantly (*p* < 0.05) and positively (*r* > 0) with the assessment of the development of knowledge, practical skills, and social skills. Similarly, the score on the A-H scale correlated significantly (*p* < 0.05) and positively (*r* > 0) with the assessment of the development of knowledge and social skills (Table 2).

**Table 2:** Relationship between participants’ opinions on classes and the level of creativity in the cognitive and characterological domains

Parameter	A-H
	Spearman’s correlation coefficient
Assessment of knowledge development	*r* = 0.13, *p* = 0.028
Assessment of practical skills development	*r* = 0.112, *p* = 0.059
Assessment of social skills development	*r* = 0.119, *p* = 0.045
**Parameter**	**C-N**
	**Spearman’s correlation coefficient**
Assessment of knowledge development	*r* = 0.173, *p* = 0.003
Assessment of practical skills development	*r* = 0.206, *p* < 0.001
Assessment of social skills development	*r* = 0.203, *p* = 0.001

**p** - statistical value, **r** - Spearman’s rank correlation coefficient, **C-N** - conformity-nonconformity, **A-H** - algorithmic-heuristic behaviors, Source: authors’ analysis

The scores on the C-N and A-H scale continua correlated significantly (*p* < 0.05) and positively (*r* > 0) with the scores on the scale of satisfaction with the learning process and self-confidence (Table 3).

**Table 3:** Relationship between nursing students’ satisfaction with the learning process and self-confidence and their creativity scores in the cognitive and characterological domains

SSCL	A-H
	Spearman’s correlation coefficient
Satisfaction with the learning process	*r* = 0.254, *p* < 0.001
Self-confidence in the learning process	*r* = 0.317, *p* < 0.001
**SSCL**	**C-N**
	**Spearman’s correlation coefficient**
Satisfaction with the learning process	*r* = 0.258, *p* < 0.001
Self-confidence in the learning process	*r* = 0.343, *p* < 0.001

**p** - statistical value, **r** - Spearman’s rank correlation coefficient, **C-N** - conformity-nonconformity, **A-H** - algorithmic-heuristic behaviors, Source: authors’ analysis

The score on the A-H scale correlated significantly (*p* < 0.05) and negatively (*r* < 0) with stress caused by the awareness that the procedure must be performed without the instructor’s assistance (*r*= -0.19; *p* = 0.001), stress related to communication with the patient and their family (*r*= -0.297; *p* < 0.001), performing the procedure itself (*r*= -0.248; *p* < 0.001) (Table 4).

The score on the C-N scale correlated significantly (*p* < 0.05) and negatively (*r* < 0) with stress caused by the awareness that the procedure must be performed without the instructor’s assistance (*r*= -0.186; *p* = 0.002), stress related to communication with the patient and their family (*r*= -0.175; *p* = 0.003), performing the procedure itself (*r*= -0.186; *p* = 0.003), and being in an unfamiliar room (*r*= -0.123; *p* = 0.038) (Table 4).

**Table 4:** Relationship between factors generating stress during simulation and the level of creativity in the cognitive and characterological domains

Parameter	A-H
	Spearman’s correlation coefficient
Stress caused by the awareness that the procedure must be performed independently	*r*=-0.19, *p* = 0.001
Stress caused by communication with the patient or their family	*r*=-0.297, *p* < 0.001
Stress caused by performing the procedure	*r*=-0.248, *p* < 0.001
Stress caused by conversation with the instructor during debriefing	*r*=-0.092, *p* = 0.12
Stress caused by being in an unfamiliar room	*r*=-0.028, *p* = 0.634
**Parameter**	**C-N**
	**Spearman’s correlation coefficient**
Stress caused by the awareness that the procedure must be performed independently	*r*=-0.186, *p* = 0.002
Stress caused by communication with the patient or their family	*r*=-0.175, *p* = 0.003
Stress caused by performing the procedure	*r*=-0.176, *p* = 0.003
Stress caused by conversation with the instructor during debriefing	*r*=-0.097, *p* = 0.1
Stress caused by being in an unfamiliar room	*r*=-0.123, *p* = 0.038 *

**p** - statistical value, **r** - Spearman’s rank correlation coefficient, **C-N** - conformity-nonconformity, **A-H** - algorithmic-heuristic behaviors, Source: authors’ analysis

## Discussion

The assessment of creativity in educational research remains challenging, particularly because its most direct indicator - creative output - is often difficult to capture in a standardized academic setting. This issue is especially relevant in nursing education, where learners differ not only in knowledge and technical performance, but also in personal resources that may shape how they engage with simulation-based learning.

From an evidence-based nursing education perspective, the present findings suggest that creativity may be considered a meaningful learner-related resource associated with the educational experience of high-fidelity simulation. Although HFS is widely recognized as an effective instructional method, its benefits are not uniform across learners. The observed associations between higher creative dispositions, greater satisfaction and self-confidence, and lower stress suggest that individual learner characteristics may be important in shaping how students experience and evaluate simulation-based education. The findings only partially supported the initial assumptions. The expectation that first-year nursing students would predominantly present a conformist attitude with a tendency toward algorithmic behaviors was not fully confirmed, as the results indicated an average level of creativity accompanied by selected pro-creative tendencies. At the same time, the findings supported the assumptions that higher creativity would be associated with greater satisfaction, higher self-confidence, and more favorable evaluations of knowledge, practical skills, and social competence development during HFS. They also supported the assumption that higher creativity may be associated with lower stress in the context of simulation. A more interpretive reading of these findings suggests that creativity may be relevant to simulation-based learning not only because of its association with positive educational outcomes, but also because of the mechanisms through which students respond to uncertainty and task demands. Higher cognitive creativity may be associated with lower stress during independent procedure performance because students with greater flexibility of thinking may be better able to generate alternative responses, reorganize their actions when difficulties arise, and tolerate the temporary absence of instructor support. In turn, higher characterological creativity may be associated with lower stress in unfamiliar settings because traits such as autonomy, openness, and relative independence from external structure may support more adaptive functioning in novel environments. From a practical perspective, the finding that students presented an average rather than low level of creativity may be interpreted as encouraging, as it suggests that creativity-related resources are present in this population and may potentially be further supported through simulation design, reflective debriefing, learner-centered facilitation, and educational strategies that encourage flexibility, problem-solving, and psychologically safe exploration of alternative approaches.

The overall pattern of results suggests that the students were not characterized by a clearly reproductive orientation, but rather by a mixed profile that included openness to understanding, reflection, and independent thinking. This may be educationally important, particularly in early nursing education, where students are still developing their preferred ways of learning and acting in demanding situations. In this sense, the present findings may indicate that even at an early stage of training, students possess creative resources that could potentially be further supported in simulation-based learning. These observations are consistent with Popek’s concept, which emphasizes that creative functioning depends on the coexistence of cognitive and personality-related dispositions. Within this framework, heuristic tendencies and nonconformity may be linked with more independent, reflective, and flexible engagement with learning tasks. At the same time, such tendencies should not be interpreted uncritically as universally advantageous, as they may also be associated with greater independence from external structure and expectations. Nevertheless, in the context of HFS, where students are exposed to dynamic and partially unpredictable situations, these dispositions may be particularly relevant [10].

It should also be noted that the literature directly examining the relationship between creativity and simulation-based learning outcomes in nursing education remains limited. For this reason, the present findings should not be interpreted as confirming a well-established and uniform pattern of relationships. Rather, they contribute to an emerging area of inquiry in which further studies are needed to determine whether these associations remain consistent across different educational settings, learner groups, and simulation designs. This cautious interpretation also appears justified in light of the broader simulation literature, where the effects of simulation-based interventions vary across studies and the quality of evidence is not always high [30].

The findings also align with previous studies suggesting that creative dispositions are not uniformly distributed in student populations and that average rather than highly pronounced creative profiles may be expected in educational settings. In this respect, the present results appear broadly consistent with earlier findings indicating that students often represent a mixed pattern of reproductive and creative tendencies rather than clearly polarized profiles. From an educational perspective, this may be regarded as encouraging, because it suggests that creativity-related resources are present and potentially available for further development rather than absent.

Another important observation is that students with more pronounced creative tendencies tended to report more positive educational experiences during HFS. This may suggest that creativity is associated not only with how students solve problems, but also with how they interpret and engage with challenging learning situations. One possible explanation is that students with stronger heuristic and nonconformist tendencies may be better able to tolerate ambiguity, engage actively with unexpected developments, and treat simulation as an opportunity for exploration rather than merely as a task requiring correct performance. Such a mechanism may help explain why creativity was associated with greater satisfaction and self-confidence in the learning process. This interpretation is also compatible with previous literature indicating that creativity-supportive educational environments should provide opportunities for active learning, reflection, practical application, and the generation of ideas [8]. In this light, HFS may be viewed as a context in which creativity-related learner characteristics become particularly visible, because simulation combines action, reflection, uncertainty, and interpersonal demands. However, given the cross-sectional design of the study, these findings should be interpreted as associations rather than as evidence that HFS increases creativity or that creativity leads to better educational outcomes.

The relationship between creativity and stress during HFS is also noteworthy. Students with higher creativity scores reported lower levels of selected stress-related experiences, particularly in relation to independent task performance, communication, and procedural execution. This may suggest that creativity is associated with greater adaptive flexibility in situations that require action under pressure. One possible explanation is that students with higher cognitive creativity may more easily generate alternative responses and reorganize their thinking in unfamiliar or demanding situations, and may therefore report less tension when instructor support is reduced. Similarly, students with stronger characterological creativity may be more comfortable acting independently and entering unfamiliar environments, which may help explain the lower stress observed in relation to selected aspects of simulation participation. These interpretations are partly supported by previous research showing that simulation may evoke psychological stress even when it is educationally beneficial [31]. Stress may be considered a relatively common component of HFS rather than merely an incidental feature. In this context, creativity may be understood as one of the learner-related resources associated with more adaptive functioning during simulation. At the same time, this interpretation should remain cautious, as the present design does not allow conclusions about causality or directionality. From a practical perspective, the findings suggest that simulation educators should remain attentive to individual differences in how students approach complex and uncertain learning situations. If creativity is associated with more positive educational experiences and lower stress, then simulation design and facilitation may benefit from incorporating elements that support reflective thinking, flexibility, active problem-solving, and psychological safety. In particular, structured debriefing, reflective questioning, supportive feedback, and learner-centered facilitation that encourages multiple approaches to clinical problem-solving while maintaining patient safety may be especially valuable. Additional structure and guidance may be particularly helpful for students who are less comfortable with ambiguity or unfamiliar situations. Previous reports have also suggested that humor, games, and peer teaching may help create a more creativity-supportive atmosphere [32].

The present study has several strengths. It addresses a relatively underexplored aspect of simulation-based nursing education by examining both cognitive and characterological dimensions of creativity in relation to satisfaction, self-confidence, perceived competence development, and stress during HFS. It also contributes to the literature by applying Popek’s concept in the context of undergraduate nursing education and by considering creativity as a learner-related resource rather than solely as a general psychological trait.

However, several limitations should be acknowledged. First, the use of KAHN III may limit the international interpretability of the findings because concepts of creativity differ across scientific traditions. Second, the cross-sectional design does not allow causal conclusions. Third, the use of convenience sampling limits the generalizability of the findings. Fourth, the study was based on self-report measures, which reflect participants’ subjective perceptions rather than objective indicators of creativity or performance. Finally, the psychometric limitations of the KAHN III scales should also be considered when interpreting the findings. Although the internal consistency of the A-H subscale was acceptable in the present sample (α = 0.729), the C-N subscale showed a borderline value (α = 0.687), which is close to the reliability reported by the scale authors. Therefore, the findings should be interpreted with appropriate caution.

Future studies could employ longitudinal designs, include participants from multiple academic centers, and complement self-report measures with more practice-oriented or observational indicators of creative functioning in simulation. Qualitative research may also help clarify how students with different creativity profiles experience the academic and simulation environment and which educational conditions are most conducive to the constructive expression of creative potential.

## Conclusions

1. The present study demonstrates that nursing students exhibited an average level of creative disposition, with selected pro-creative tendencies visible in both characterological and cognitive domains.
2. Higher levels of creativity in both the cognitive and characterological domains were associated with greater satisfaction and self-confidence during high-fidelity simulation sessions, indicating that creativity functions as a learner-related factor linked to positive educational experiences.
3. Creative dispositions were also related to more favorable perceptions of selected learning outcomes, including knowledge, practical skills, and social competence development.
4. From an evidence-based nursing education perspective, creativity may be understood as a learner-related resource associated with lower stress in selected areas of high-fidelity simulation, including independent task performance, patient communication, and procedural execution.
5. The findings suggest that creativity may be considered an important learner-related factor in undergraduate nursing education. Incorporating creativity-supportive elements into the design and delivery of high-fidelity simulation may be worth considering when developing learner-centered and evidence-informed educational strategies.

## Electronic Supplementary Material

Below is the link to the electronic supplementary material.

Supplementary Material 1

## Data Availability

The datasets used and/or analysed during the current study are available from the corresponding author on reasonable request.

## Abbreviations

CIEM: Centre for Innovative Medical Education
HFS: High-fidelity Simulation
KAHN III: Creative Behavior Questionnaire
OECD: Organisation for Economic Co-operation and Development
SSCL: Student Satisfaction and Self-Confidence in Learning Scale
