# Supplementary material for: Creativity in high-fidelity simulation-based nursing education: associations with learning outcomes and stress
Source: BMC Nurs. 2026 May 15;25:606. doi: 10.1186/s12912-026-04751-4 (PMC13352980; doi:10.1186/s12912-026-04751-4)
Supplement: Supplementary file 1 — Supplementary Material 1 [file 12912_2026_4751_MOESM1_ESM.docx]

**ORIGINAL SURVEY QUESTIONNAIRE**

Please respond to the statements given by ticking one number that reflects your opinion (a legend is given below) or write your own text in the space provided.

Response scale:

1 - definitely not

2 - rather not

3 - neither yes nor no

4 - rather yes

5 - definitely yes

| **Questions** | | **Scale of responses** | | | | |
| --- | --- | --- | --- | --- | --- | --- |
| 1. Do classes at the Centre for Innovative Medical Education in Fundamentals of Nursing: | were of interest to you? | 1 | 2 | 3 | 4 | 5 |
|  | enhance your knowledge? | 1 | 2 | 3 | 4 | 5 |
|  | influenced the development of practical skills? | 1 | 2 | 3 | 4 | 5 |
|  | influenced the development of social skills, i.e. the establishment of relationships with the patient and/or his/her family? | 1 | 2 | 3 | 4 | 5 |
| 1. Did the debriefing carried out (interview with the teacher after the scenario was carried out) provided answers to the questions: | what was done well? | 1 | 2 | 3 | 4 | 5 |
|  | what should be improved? | 1 | 2 | 3 | 4 | 5 |
|  | how should any errors/failures be corrected? | 1 | 2 | 3 | 4 | 5 |
|  | provided an adequate summary of the activities carried out? | 1 | 2 | 3 | 4 | 5 |
| 1. How would you rate your attitude in class? | I was involved during the implementation of the scenario | 1 | 2 | 3 | 4 | 5 |
|  | I was confused during the implementation of the scenario | 1 | 2 | 3 | 4 | 5 |
| 1. What generated stress during the class? | awareness that the procedure should be carried out without the assistance of an instructor | 1 | 2 | 3 | 4 | 5 |
|  | communication with the patient or his/her family | 1 | 2 | 3 | 4 | 5 |
|  | implementation of the procedure | 1 | 2 | 3 | 4 | 5 |
|  | discussion with the teacher during debriefing | 1 | 2 | 3 | 4 | 5 |
|  | being in a room that I was not familiar with | 1 | 2 | 3 | 4 | 5 |

Sociodemographic data

Gender:

1. Female
2. Male

Age: …………………………………..
